# The Effect of Balance and Sand Training on Postural Control in Elite Beach Volleyball Players

**DOI:** 10.3390/ijerph17238981

**Published:** 2020-12-02

**Authors:** Sergio Sebastia-Amat, Luca Paolo Ardigò, Jose Manuel Jimenez-Olmedo, Basilio Pueo, Alfonso Penichet-Tomas

**Affiliations:** 1 Physical Education and Sport Sciences, Faculty of Education, University of Alicante, 03690 Alicante, Spain; sergio.sebastia@ua.es (S.S.-A.); j.olmedo@ua.es (J.M.J.-O.); alfonso.penichet@ua.es (A.P.-T.); 2 Department of Neurosciences, Biomedicine and Movement Sciences, School of Exercise and Sport Science, University of Verona, 37131 Verona, Italy; luca.ardigo@univr.it

**Keywords:** athletes, posturography, core, stability, proprioception

## Abstract

The aim of this work was to evaluate the effectiveness of a 12-week-long balance training program on the postural control of elite male beach volleyball players and the effect on balance when swapping to specific sports training in the sand in the following 12 weeks. Six elite players were tested before and after the balance training program and also 12 weeks after the balance training had finished. To this aim, a pressure platform was used to collect the following center of pressure parameters: path length, speed, mean position, and root-mean-square amplitude in the medial-lateral and anteroposterior planes. Romberg quotients for the center of pressure parameters were also calculated. The results of the present study showed better static postural control after specific balance training: smaller path length and speed under open eyes condition in dominant (*p* = 0.015; *p* = 0.009, respectively) and non-dominant monopedal stances (*p* = 0.005; *p* = 0.004, respectively). Contrastingly, 12 weeks after the balance training program, the path length and speed values under open eyes condition in bipedal stance increased significantly (*p* = 0.045; *p* = 0.004, respectively) for sand training. According to our results, balance training is effective to achieve positive balance test scores. It is speculated, and yet to be proven, that sand training could be effective to improve dynamic and open eyes postural control during beach volleyball practice. In beach volleyball players, a balance training program is effective to develop static balance but the effect of ecological sand training on dynamic performance deserves specific investigation.

## 1. Introduction

Postural control is a complex function of the central nervous system for detecting sensory stimuli, interpreting information, and responding appropriately in order to maintain an upright position [1,2]. The development of postural control requires integration between sensory systems, i.e., vestibular, visual, and somatosensory systems [3,4]. The two main functional objectives of postural control are postural orientation and postural equilibrium [5].

Postural control depends not only on the health state but also on training capacity through variations in the tone of postural muscles [6,7]. Thus, physical training allows for the acquisition of new skills and strategies for postural control improvements [8]. Balance training is widely used in rehabilitation, sports, and injury prevention programs [9]. Likewise, balance training has been shown to be effective for the improvement of postural control in different populations: active and inactive young people [10,11], athletes [12,13,14], and the elderly [15,16].

In the sports field, an improvement in postural control is associated with better performance [17], mainly due to an increase in the efficiency of sport-specific actions [18], better force production [19], and reduction of injury risk [20]. Although balance training seems to be effective in improving performance only in specific trained tasks [21], many mechanisms related to the plasticity of postural control and the sport experience are not yet understood [18,22].

There are limited data on the benefits of balance training in elite athletes [18] especially when athletes play on unstable surfaces such as sandy courts, where physiological and biomechanical characteristics differ from firm ground [23,24,25]. Despite studies addressing balance training in volleyball [12,26,27,28], no study related to beach volleyball has been found. Therefore, it would be interesting to explore the effect of balance training in a discipline played on unstable surfaces [29], where demands of motor control are higher than on stable surfaces [30]. In the same way, it would be useful to know if sand training can be a substitute for classical balance training in elite beach athletes with advanced motor skills to maintain balance.

The present study aimed to investigate whether a balance training program could improve postural control in male elite beach volleyball players. It was hypothesized that stabilometric variables would be enhanced as a consequence of the balance training program, mainly decreasing total excursion (TE) and speed, and that these variables would return to baseline values after the replacement of balance training with specific sports training. To this end, the effect of balance training, elimination, and replacement for specific sports training in the sand were studied with regards to postural control.

## 2. Materials and Methods

### 2.1. Participants

We studied three elite international beach volleyball teams, six players (age 23.6 ± 3.3 years, height 188.2 ± 7.9 cm, body mass 77.7 ± 13.1 kg, BMI 22.3 ± 2.6 kg/m^2^, body fat 8.9 ± 2.5% (measured with a Tanita BC-545N body composition analyzer), professional experience 4.9 ± 1.8 years, predominance of arm and leg, right and left, 6/0). Three players played in the blocker position and the other three played in the defense position. The requirements to participate in the present study were to have competed in the national winter league and the National Circuit “Madison Beach Volley Tour” for three years, to have competed during at least the last year in international championships, and previous experience with balance training. A power analysis using G*3-Power (Heinrich Heine Universität, Düsseldorf, Germany) [31] indicated that this sample size provided 80% power to detect effects of *d* = 0.6 (medium effect) in a repeated measurement test with α = 0.05. The Ethics Committee at the University of Alicante gave institutional approval to this study, in accordance with the Declaration of Helsinki (UA-2018-12-19), with the consent of athletes duly informed of the procedure and parts of the study.

### 2.2. Measures

A baropodometric platform (FreeMed, Rome, Italy) was used for the stabilometric analyses, with an active surface of 400 × 400 mm, 8 mm thickness and a sample frequency of 100 Hz. The test was carried out three times with two minutes rest between trials. Stabilometric parameters were assessed three times and the average value was registered. The reference stabilometric values to express deviation of the center of pressure (CoP) were: path length, defined as the length of the total distance of the CoP over the course of the trial duration; speed, defined as total distance traveled by the CoP over time. It is suggested that increases in TE represent a decreased ability by the postural control system to maintain balance. Similarly, increases in CoP velocity could indicate a decreased ability to control posture [32]. Four displacement parameters were also measured regarding the average absolute displacements around the mean CoP: medial-lateral plane, anteroposterior plane, root-mean-square amplitude in medial-lateral plane, and root-mean-square amplitude in anteroposterior plane. The Romberg test was measured for 30 s in the following positions: bipedal stance with eyes open, bipedal stance with eyes closed, dominant monopedal stance with eyes open, dominant monopedal stance with eyes closed, non-dominant monopedal stance with eyes open, and non-dominant monopedal stance with eyes closed [14,33,34]. Traditional Romberg quotients for the CoP parameters were also calculated (eyes closed/eyes open) [35] as an indicator of proprioceptive and visual field contribution to postural stability [36,37]. A Romberg quotient higher than 1.0 denotes a greater postural sway during eyes closed condition [35]. The test order (positions and deprivation conditions) were randomized.

Data collection was initiated after the players were stable in an erect position on the baropodometric platform. The players remained barefoot and positioned with the feet facing forward. The distance between feet was considered according to the hip width. The arms remained relaxed and parallel to the body and the head faced forward. The players were asked to remain in this position during the evaluation process.

### 2.3. Design and Procedures

An alternating treatment design was used for the present study to explore the alternation of two interventions (balance and sand training) with different proprioceptive stimuli. In this between-series design, the analysis was focused on the comparison of treatment condition outputs [38]. All measurements were taken during the in-season phase. During the pre-season phase (6 weeks) the players had 5 weekly sessions in the sand and 5 weekly sessions in the gym. The volume and intensity of training sessions increased over the weeks. No specific balance training was carried out in this phase.

The competition phase schedule was as follows: 6 weekly sessions in the sand with an approximate duration of 120 min each. Of these, days 1, 3, 5, and 6 were completely devoted to technical and tactical training. The first hour of day 2 and day 4 was devoted to specific physical training and the second hour was also devoted to technical and tactical training. In the same way, there were also 4 weekly sessions of training in the gym with an approximate duration of 120 min each.

**Figure 1:**
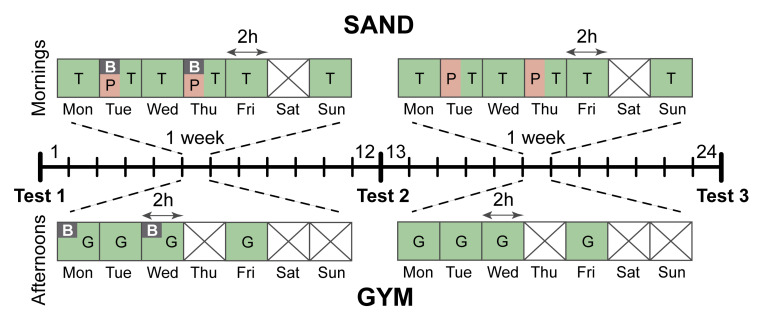
Experimental procedure showing weekly sessions for 24 weeks. Test 1: baseline; Test 2: post-balance training; Test 3: post-sand training. T: technical and tactical training, B: balance training, P: physical training, G: gym training. Days without training are displayed with crossed squares.

The intervention began 3 weeks after the competition phase has started, so that the players could adjust to training. In the first week of intervention, all players underwent a postural control assessment, for the balance baseline level (Test 1 in Figure 1). Then, participants took part in a balance training program over 12 weeks with 48 total sessions (4 sessions per week, 2 sand sessions and 2 gym sessions, 20 min per session). During this first period, the players also completed 6 weekly sessions in the sand with an approximate duration of 120 min each. Of these, 2 weekly sessions had balance training. The first 20 min of the session after the warm-up was devoted to balance training (40 min/week). In the same way, there were also 4 weekly sessions of training in the gym. In two of them, the first 20 min were devoted to balance training (40 min/week). At the end of the intervention, the second evaluation of postural control was carried out (Test 2).

In the 12 weeks following the second period, the training volume (weekly sessions of sand and gym) was the same as in the first period. The balance training was eliminated both in the sand and in the gym and was replaced by specific physical sand training. This training consisted of a selection of similar exercises to the real game to maintain the same work volume and intensity in the sessions as in the first intervention.

Finally, a third measurement (Test 3) was made to compare the data with the two previous tests to explore if the specific work in the sand could substitute balance training with regards to the improvement of the stability of the athletes.

### 2.4. Balance Training

The conventional warm-up consisted of dynamic stretching and moderate aerobic exercises before the regular practice sessions. The time spent to perform balance training did not include the warm-up. The coach conducted the sessions and gave feedback on exercise techniques. Each 20 min session consisted of circuit training with 10 exercises (30 s work/30 s rest between exercises) and 1 min rests between circuits (two laps). Based on previous studies [17,39], the balance training consisted of performing static and dynamic exercises in which the participant’s equilibrium was more compromised and the exercises gradually progressed in complexity. In some exercises, the vision of the subject was totally or partially limited, so it was a challenge mainly for the core zone. The exercises were performed in monopedal and bipedal stances and auxiliary materials such as scales coordination, medicine balls, or unstable materials were used to create perturbations. The set of 10 exercises was as follows: bilateral squat on a BOSU ball (one for each leg) and two-hand bump using the wall; single-leg standing on inflated disk progressing to closed eyes execution; lateral medicine ball throwing on a BOSU ball (one for each leg); supine straight leg bridge on a Swiss ball; coordination scale and single leg squat; banded triplanar toe taps progressing to closed eyes execution; paloff press with a slight rotation progressing to monopedal stance; plank with elbows on a Swiss ball progressing to closed eyes execution; on a BOSU ball (one for each leg) the player used the Swiss ball to knock the beach volleyball ball to the coach; attack reception while standing with one or both legs on a BOSU ball; lunge on foam surface progressing to closed eyes execution. The main goal was to make the exercises as similar as possible to the real game actions [40], so that they could be transferred to beach volleyball.

### 2.5. Statistical Analysis

Descriptive statistics (means and standard deviations), the distribution of normality (Kolgomorov–Smirnov and Lilliefors test), and Levene homogeneity tests were calculated for each stabilometric value. A repeated-measures ANOVA was conducted to compare postural control variables between baseline level, post-balance training, and post sand with post hoc comparisons using the Bonferroni test. A value of *p* < 0.05 was used to identify statistically significant differences. The percentage variation was calculated using the following formula: (Post-Test − Pre-Test)/Pre-Test × 100. Cohen’s *d* was used as a measure of the effect size of differences between tests and interpreted according to Cohen’s thresholds as small (0.00 ≤ 0.49), medium (0.50 ≤ 0.79), and large (≥ 0.80) [41]. Analyses were performed using the Statistical Package for Social Sciences v.22 (IBM, Armonk, NY, USA).

## 3. Results

The values of the parameters related to postural control in static bipedal stance are shown in Table 1. The results when baseline level and post-balance training were compared in open eyes condition showed an improvement in the main indicators of postural control after the balance training intervention: 29.4% path length (*p* = 0.240) and 28.7% speed (*p* = 0.241) decreases. The improvements of these variables meant that the participants were more stable and efficient in controlling their posture. Significant worsening for the same variables, i.e., 38.6% path length (*p* = 0.045) and 38.7% speed (*p* = 0.004) increases, resulted between post-balance training and post-sand training, once the replacement training in sand had finished. Similar results were found in closed eyes condition although they were not statistically significant, except for root-mean-square amplitude in the antero-posterior plane (*p* = 0.038) when baseline level and post-sand training were compared.

Test 3 reported displacements of the center of pressure (CoP) in the medio-lateral and anterior-posterior directions (X and Y mean variables) closer to zero compared with previous tests. This meant that participants had shorter excursions in both planes, especially in the anterior-posterior plane during open eyes condition.

The comparison dominant monopedal variables shown in Table 2 depict significant improvements in path length (23.4% decrease, *p* = 0.015) and speed (30.5% decrease, *p* = 0.009) in open eyes condition for baseline level and post-sand training, which suggest an improvement in postural control after the intervention of a balance training program. As in the previous section, the results got worse once the substitute intervention in the sand was completed. In conditions of visual deprivation, there were improvements in the main indicators of stability after the balance training program and a slight worsening once the replacement intervention in the sand was finished.

The results of the non-dominant monopedal stance (Table 3) also showed an improvement in the main variables related to postural control with open eyes after the intervention of a balance training program: improvement in path length (22.5% decrease, *p* = 0.005) and speed (25.7% decrease, *p* = 0.004). Similar to the bipedal stance, deteriorations in open eyes conditions can be observed after completion of the sand training. A comparison of baseline level and post-balance training in closed eyes conditions showed an improvement in the main variables related to postural control after the intervention of a balance training program.

The Romberg quotient related to bipedal stance showed a significant increase between baseline level and post-balance training for the path length (*p* = 0.024) and mean speed (*p* = 0.023). On the other hand, the Romberg quotient related to non-dominant monopedal stance showed a significant increase between post-balance training and post-sand training for the path length (*p* = 0.019) and mean speed (*p* = 0.013).

Tests 2 and 3 reported displacements in the center of pressure (CoP) in the medio-lateral and anterior-posterior directions (X and Y mean variables) closer to zero compared with Test 1. These improvements were only significant for closed eyes conditions.

**Figure 2:**
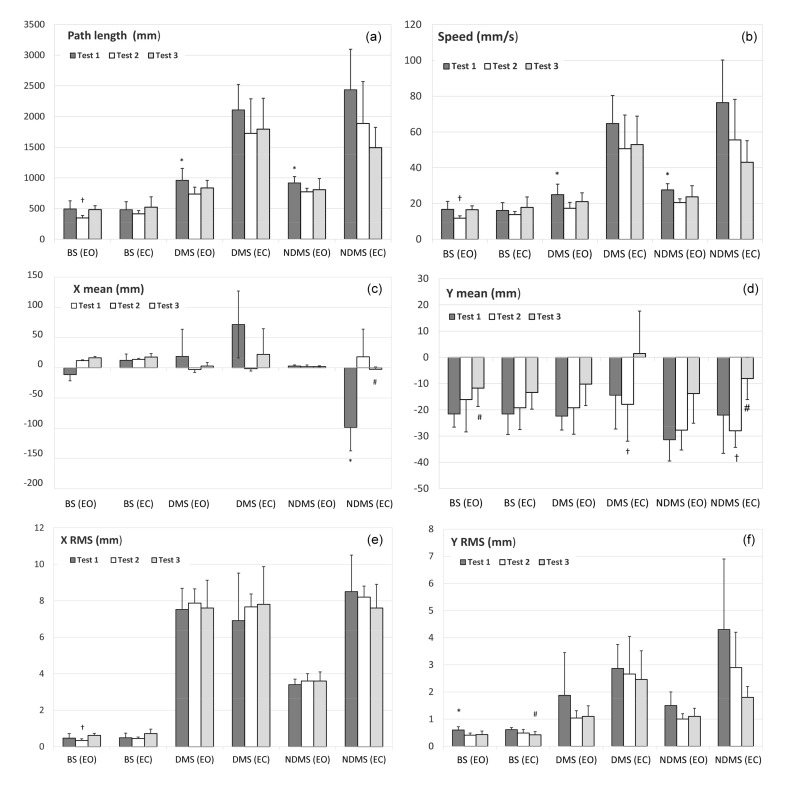
Stabilometric variables compared for bipedal stance (BS), dominant monopedal stance (DMS), and non-dominant monopedal stance (NDMS) for eyes open (EO) and eyes closed (EC) in baseline (Test 1), post-balance (Test 2) and post-sand (Test 3) tests. (**a**) Path length, (**b**) Speed, (**c**) X mean, (**d**) Y mean, (**e**) X RMS and (**f**) Y RMS. * = significant difference between Test 1 and 2; † = significant difference between Test 2 and 3; # = significant difference between Test 1 and 3. Significance: *p* < 0.05.

Figure 2 shows the results of the three tables in a graphical way to help the reader visualize the variations in the variables under examination for the three tests.

## 4. Discussion

The present study was designed to evaluate the effect of a 12-week-long balance training program on the postural control of elite male beach volleyball players and the effect on balance when swapping to specific sports training in sand in the 12 weeks following the program. The results of the present study showed an improvement in postural control, mainly in the monopedal stance, after the implementation of a balance training program. Subsequently, a worsening in postural control values was reported after the elimination and replacement of the balance training. The reference variables such as TE and speed decreased after balance training (BT) intervention, both in bipedal and monopedal stances, although improvements were only significant in the monopedal stance for the open eyes condition. Despite non-significant improvements being found in bipedal stance after BT intervention, it was reported that this condition significantly worsened after the elimination of the BT program. Subsequently, a worsening in postural control values was reported after the elimination and replacement of the balance training.

The ability to maintain or recover balance in any sport is necessary for the correct performance of specific actions [28]. This ability has a relevant role in the performance of volleyball actions, either in contact with the ground (approach to the net, receptions, and placements) or in the air phase (attack and blocking) [42,43]. Several meta-analyses based on the effects of balance training [9,11,17,44,45] revealed that the majority of studies reported a positive effect on postural control.

Consistent with the previous meta-analysis, our results showed an improvement in postural control after an intervention based on balance training. The results indicated an improvement between baseline level and post-balance training in the path length and speed variables, for both eyes open and eyes closed conditions, although improvements were only significant for the open eyes condition. This can be explained by the improvement in both conditions, especially in open eyes conditions, possibly due to the greater volume of training carried out under this condition. In the same way, the Romberg quotients for these variables increased significantly between baseline level and post-balance training in bipedal stances, an indicator of a major contribution of the visual field to postural stability. These findings are in line with the meta-analysis of Kümmel et al. [21], in which the effectiveness of balance training was shown only in those activities carried out under the same training conditions. Similarly, some studies [34,46] comparing different groups of athletes reported improvements mainly for open eyes conditions, suggesting the importance of specificity of training and the stimulation of proprioceptive channels in postural control.

Considering the stance conditions, the improvements found were only significant in dominant and non-dominant monopedal stances for the TE and speed variables. This may be because most actions in volleyball and beach volleyball require high motor control in standing conditions [12,28], so an increase of training in the monopedal stance can be a sufficient stimulus to produce adaptations. Likewise, the fact that there were more parameters enhanced in the non-dominant monopedal stance may be due to the muscular reinforcement of the weakest limb as a consequence of the training program [12].

The results of our study were mainly compared with the study of Pau et al. [12], who reported a potential effect of balance training in postural control of female volleyball players. Conversely to our results, the improvements were obtained for the closed eyes condition. They obtained significant results on sway for both bipedal and non-dominant monopedal stances. However, non-significant results were found in the dominant monopedal stance.

On the other hand, the results of the present study showed a significant decrease in postural control values after removing the balance training program. Therefore, sport-specific training in the sand would not act as a substitute for balance training in players adapted to play on unstable surfaces. This fact was observed in non-athletic populations [47,48] where a process of detraining or loss of postural control could be observed after a period of inactivity or cessation of the stimulus. In the sports field, Dai et al. [27] studied the process of detraining in volleyball players one month after the end of the season. As in the present study, the average speed increased and the CoP speed parameters tended to change in all directions, suggesting a decrease in postural control. Nevertheless, studies that relate postural control and detraining have limitations mainly due to sample characteristics and detraining modality. It should be noted that during the post-sand period, displacements of the center of pressure (CoP) in the medio-lateral and anterior-posterior directions (X and Y mean variables) were closer to zero. This could reveal a specific adaptation to sand training, questioning the real transfer of BT to the beach volleyball balance performance due to the high level of specificity of this training [21]. Therefore, these results could be interpreted as a worsening of postural control for the general population although they could also be due to specific adaptations for beach volleyball sport practice.

In conclusion, the BT program improved the postural control of beach volleyball players. The elimination and replacement of specific beach volleyball training apparently could be interpreted as a worsening of postural control. However, specific adaptations to beach volleyball should not be entirely discarded. Further investigation is needed to investigate the effect of BT on beach volleyball balance performance.

A limitation of the present study is that its participants were all male, viz., with a body center of mass on average higher than females, which could influence different gender balance strategies. Involving females as well would allow larger generalizations of the findings on the whole. Furthermore, it can only be supposed that a sport-specific sand training could effectively develop the dynamic and open eyes postural control acknowledged as required for successful beach volleyball performance. The present study did not specifically investigate this. Therefore, the effect of ecological sand training on dynamic performance should be the object of further research in beach volleyball players. The transfer of dynamic balance management to performance remains unknown and needs to be evaluated during real matches on sand.

## 5. Conclusions

The results of the present study show that a balance training program in beach volleyball players is effective for acquiring proper static balance control. It was also shown that the same players undergoing their habitual and ecological beach volleyball sand training experienced worsening of their static balance control. It can be hypothesized that beach volleyball sand training might be selectively effective in acquiring a capability different from static balance control, namely a dynamic and open eyes postural control during beach volleyball practice. Such a capability might be acquired at the price of a worsening of static balance control scores. Further studies should investigate relationships between sand training and beach volleyball performance. To date, it can only be hypothesized that beach volleyball players aiming at conditioning specific postural control abilities such as dynamic and open eyes postural balance should favor sand training over gym training.

## Figures and Tables

**Table 1:** Bipedal stance. Parameters expressed as mean ± SD.

Variable and Condition	Test 1: Baseline	Test 2: Post-Balance	Test 3: Post-Sand	Test 1 vs. 2 Cohen’s d	Test 2 vs. 3 Cohen’s d	Test 1 vs. 3 Cohen’s d
Path length (mm)						
Eyes open	493 ± 132	348 ± 38 ^†^	483 ± 64	1.5 (large)	2.6 (large)	0.1 (small)
Eyes closed	481 ± 129	413 ± 53	524 ± 167	0.7 (medium)	0.9 (large)	0.3 (small)
Romberg	0.98 ± 0.14 *	1.19 ± 0.11	1.10 ± 0.38	1.5 (large)	0.3 (small)	0.3 (small)
Speed (mm/s)						
Eyes open	16.7 ± 4.48	11.8 ± 1.29 ^†^	16.5 ± 2.16	1.5 (large)	2.6 (large)	0.1 (small)
Eyes closed	16.1 ± 4.37	13.8 ± 1.73	17.8 ± 5.85	0.7 (medium)	0.9 (large)	0.3 (small)
Romberg	0.97 ± 0.14 *	1.17 ± 0.10	1.09 ± 0.39	1.6 (large)	0.3 (small)	0.4 (small)
X Mean (mm)						
Eyes open	−11.5 ± 10.1	−10.6 ± 8.9	−8.0 ± 3.6	0.1 (small)	0.4 (small)	0.5 (small)
Eyes closed	−12.2 ± 10.6	−13.1 ± 6.9	−8.9 ± 4.7	0.1 (small)	0.7 (medium)	0.4 (small)
Y Mean (mm)						
Eyes open	−21.6 ± 5.0	−16.1 ± 12.3	−11.7 ± 7.0 ^#^	0.6 (medium)	0.4 (small)	1.6 (large)
Eyes closed	−21.6 ± 7.8	−19.2 ± 8.3	−13.4 ± 6.3	0.3 (small)	0.8 (medium)	1.2 (large)
X RMS (mm)						
Eyes open	0.47 ± 0.25	0.34 ± 0.09 ^†^	0.62 ± 0.11	0.7 (medium)	2.8 (large)	0.8 (medium)
Eyes closed	0.49 ± 0.25	0.45 ± 0.08	0.72 ± 0.24	0.2 (small)	1.5 (large)	0.9 (large)
Y RMS (mm)						
Eyes open	0.60 ± 0.12 *	0.41 ± 0.08	0.43 ± 0.13	1.9 (large)	0.2 (small)	1.4 (large)
Eyes closed	0.61 ± 0.08	0.49 ± 0.13	0.42 ± 0.12 ^#^	1.1 (medium)	0.6 (medium)	1.9 (large)

X Mean: mean position in medial-lateral plane; Y Mean: mean position in antero-posterior plane; X RMS: root-mean-square amplitude in medial-lateral plane; Y RMS: root-mean-square amplitude in the antero-posterior plane; (+/−) sign indicate the direction on X and Y axis; * = significant difference between Test 1 and 2; ^†^ = significant difference between Test 2 and 3; ^#^ = significant difference between Test 1 and 3. Significance: *p* < 0.05.

**Table 2:** Dominant monopedal stance. Parameters expressed as mean ± SD.

Variable and Condition	Test 1: Baseline	Test 2: Post-Balance	Test 3: Post-Sand	Test 1 vs. 2 Cohen’s d	Test 2 vs. 3 Cohen’s d	Test 1 vs. 3 Cohen’s d
Path length (mm)						
Eyes open	960 ± 194 *	736 ± 113	837 ± 122	1.41 (large)	0.9 (large)	0.8 (medium)
Eyes closed	2107 ± 415	1725 ± 565	1794 ± 503	0.77 (medium)	0.1 (small)	0.7 (medium)
Romberg	2.24 ± 0.48	2.37 ± 0.84	2.20 ± 0.73	0.19 (small)	0.2 (small)	0.1 (small)
Speed (mm/s)						
Eyes open	24.9 ± 5.9 *	17.3 ± 3.3	21.0 ± 4.9	1.58 (large)	0.9 (large)	0.7 (medium)
Eyes closed	64.7 ± 15.7	50.6 ± 18.8	52.9 ± 15.9	0.81 (large)	0.1 (small)	0.7 (medium)
Romberg	2.68 ± 0.70	2.99 ± 1.24	2.67 ± 1.11	0.31 (small)	0.3 (small)	0.0 (small)
X Mean (mm)						
Eyes open	18.8 ± 44.9	−2.78 ± 4.89	−2.77 ± 5.97	0.50 (small)	0.0 (small)	0.5 (medium)
Eyes closed	71.5 ± 55.4	−1.45 ± 4.08	22.2 ± 42.5	1.78 (large)	0.7 (medium)	1.0 (large)
Y Mean (mm)						
Eyes open	−22.4 ± 5.3	−19.2 ± 10.1	−10.2 ±8.2	0.40 (small)	1.0 (large)	1.8 (large)
Eyes closed	−14.4 ± 12.9	−17.9 ± 14.1 ^†^	1.43 ± 16.15	0.26 (small)	1.1 (large)	0.9 (large)
X RMS (mm)						
Eyes open	7.52 ± 1.16	7.87 ± 0.78	7.60 ± 1.53	0.35 (small)	0.2 (small)	0.1 (small)
Eyes closed	6.91 ± 2.61	7.66 ± 0.71	7.80 ± 2.07	0.39 (small)	0.1 (small)	0.4 (small)
Y RMS (mm)						
Eyes open	1.88 ± 1.57	1.04 ± 0.27	1.10 ± 0.39	0.75 (medium)	0.2 (small)	0.7 (medium)
Eyes closed	2.87 ± 0.88	2.66 ± 1.39	2.46 ± 1.06	0.18 (small)	0.2 (small)	0.4 (small)

X Mean: mean position in medial-lateral plane; Y Mean: mean position in antero-posterior plane; X RMS: root-mean-square amplitude in medial-lateral plane; Y RMS: root-mean-square amplitude in the antero-posterior plane; (+/−) sign indicate the direction on X and Y axis; * = significant difference between Test 1 and 2; ^†^ = significant difference between Test 2 and 3. Significance: *p* < 0.05.

**Table 3:** Non-dominant monopedal stance. Parameters expressed as mean ± SD.

Variable and Condition	Test 1: Baseline	Test 2: Post-Balance	Test 3: Post-Sand	Test 1 vs. 2 Cohen’s d	Test 2 vs. 3 Cohen’s d	Test 1 vs. 3 Cohen’s d
Path length (mm)						
Eyes open	918 ± 102 *	772 ± 59	806 ± 182	1.7 (large)	0.2 (small)	0.8 (medium)
Eyes closed	2435 ± 662	1887 ± 682	1491 ± 332	0.8 (large)	0.7 (medium)	1.8 (large)
Romberg	2.63 ± 0.53	2.66 ± 0.98 ^†^	1.94 ± 0.66	0.1 (small)	0.9 (large)	1.2 (large)
Speed (mm/s)						
Eyes open	27.6 ± 3.4 *	20.5 ± 2.1	23.7 ± 6.2	2.5 (large)	0.7 (medium)	0.8 (medium)
Eyes closed	76.4 ± 23.8	55.5 ± 22.7	43.0 ± 12.1	0.9 (large)	0.7 (medium)	1.8 (large)
Romberg	2.74 ± 0.66	2.73 ± 1.22	1.93 ± 0.76	0.0 (small)	0.8 (medium)	1.1 (large)
X Mean (mm)						
Eyes open	2.95 ± 1.70	1.78 ± 2.78	1.91 ± 1.39	0.5 (small)	0.1 (small)	0.6 (medium)
Eyes closed	−98.6 ± 39.0 *	−18.1 ± 45.8	−2.5 ± 3.8 ^#^	1.9 (large)	0.5 (small)	3.5 (large)
Y Mean (mm)						
Eyes open	−31.4 ± 8.1	−27.7 ± 7.6	−13.8 ± 11.3	0.5 (small)	1.4 (large)	1.8 (large)
Eyes closed	−22.0 ± 14.6	−28.0 ± 6.3 ^†^	−8.14 ± 8.01 ^#^	0.5 (medium)	2.8 (large)	1.2 (large)
X RMS (mm)						
Eyes open	3.44 ± 0.34	3.62 ± 0.42	3.58 ± 0.54	0.6 (medium)	0.0 (small)	0.5 (small)
Eyes closed	8.48 ± 2.00	8.16 ± 0.56	7.56 ± 1.26	0.2 (small)	0.6 (medium)	0.5 (medium)
Y RMS (mm)						
Eyes open	1.48 ± 0.49	1.02 ± 0.16	1.11 ± 0.33	1.3 (large)	0.4 (small)	1.0 (large)
Eyes closed	4.32 ± 2.63	2.93 ± 1.29	1.79 ± 0.45	0.7 (medium)	1.1 (large)	1.3 (large)

X Mean: mean position in medial-lateral plane; Y Mean: mean position in antero-posterior plane; X RMS: root-mean-square amplitude in medial-lateral plane; Y RMS: root-mean-square amplitude in the antero-posterior plane; (+/−) sign indicate the direction on X and Y axis; * = significant difference between Test 1 and 2; ^†^ = significant difference between Test 2 and 3; ^#^ = significant difference between Test 1 and 3. Significance: *p* < 0.05.
